# A Rare Case of Granulosa Cell Tumor Associated With Endometrial Carcinoma - A Case Report

**DOI:** 10.7759/cureus.31122

**Published:** 2022-11-05

**Authors:** Sonali Chauhan, Deepti Shrivastava, Neema Acharya, Kamlesh Chaudhari

**Affiliations:** 1 Obstetrics and Gynecology, Datta Meghe Institute of Medical Science, Wardha, IND; 2 Obstetrics and Gynecology, Jawaharlal Nehru Medical College, Datta Meghe Institute of Medical Science, Wardha, IND; 3 Obstetrics and Gynecology, Jawaharlal Nehru Medical College, Datta meghe institute of medical sciences, Wardha, IND

**Keywords:** case report, prognosis, staging laparotomy, granulosa cell tumor, endometrial carcinoma

## Abstract

Adult granulosa cell tumors are uncommon ovarian tumors mainly diagnosed after the age of 30, and the average age is roughly 55. Very few cases are associated with endometrial carcinoma. Most of these cases are well-differentiated endometrioid adenocarcinoma with excellent prognosis if found early. Heavy, irregular menstrual bleeding and postmenopausal bleeding are frequent and are caused by endometrial shedding due to unopposed estrogen action for a prolonged period of time.

A 65-year-old female para 4, live 4 (P4L4) came with postmenopausal bleeding for six months. Various investigations were done, which revealed endometrial cancer type 1. A staging laparotomy was performed, which showed a right hemorrhagic ovarian mass; on a frozen section, it came to be a granulosa cell tumor.

The rare association between granulosa cell tumor and endometrial carcinoma can be diagnosed with a good correlation of clinical, histopathological, and radiological findings.

## Introduction

The ovarian stroma is the origin of a diverse collection of uncommon neoplasms known as sex cord-stromal tumors (SCSTs). Granulosa cell tumors make up 70% of all ovarian SCSTs. Malignant cells are thought to have originated from those cells encircling the germinal cells within ovarian follicles. The adult form, which accounts for 95% of cases, and the juvenile type, which accounts for 5% of cases, are the two clinically and histologically distinct kinds [1-3].

Most women are diagnosed with adult granulosa cell tumors after the age of 30, and the average age is roughly 55. Heavy, irregular menstrual bleeding and postmenopausal bleeding are frequent and are caused by the endometrium being exposed to estrogen for an extended period of time. In 25 to 30 percent of individuals with adult granulosa cell tumors, concomitant disease such as endometrial hyperplasia or adenocarcinoma has been identified as being connected to this estrogen excess [3]. If an adult granulosa cell tumor is discovered during surgery, tumor markers may be required. Inhibin B appears to be more reliable than inhibin A, and it is usually raised months before a clinical diagnosis of recurrence [4-5].

We describe the case of a 65-year-old woman who had an adnexal mass, postmenopausal bleeding, and endometrial carcinoma. She also had an adult granulosa cell tumor of the ovary. Histopathological testing of the specimen was obtained after the patient underwent the total abdominal hysterectomy with bilateral salpingo-oophorectomy, infra-colic omentectomy, and pelvic lymphadenectomy. She was discharged on the 12th day after the sutures were removed and had a satisfactory post-operative recovery.

## Case presentation

**Figure 1:**
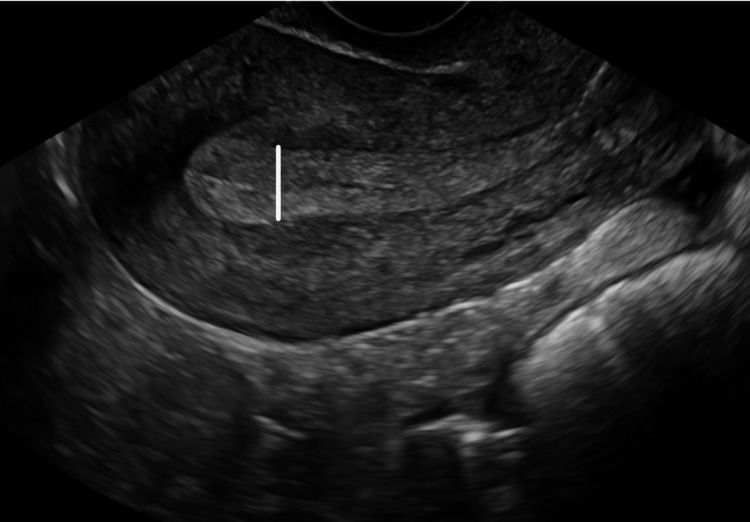
Ultrasonography showing thickened endometrium (white line)

A 65-year-old woman (para 4, live 4) with all full-term normal deliveries presented with lower abdominal pain and postmenopausal bleeding. She attained menarche at 15 years of age and menopause at 50 years of age. She is a known case of systemic hypertension diagnosed 10 years ago. On general examination, she had a visible pallor. During a gynecological examination, the uterus was bulky, and a 4 x 4 cm mobile, hard mass that was solid in consistency was felt to be anteriorly separated from the uterus. Inhibin A level was 2.21 picogram/milliliter, and inhibin B level was 16.1 picogram/milliliter. The results of abdominal ultrasound showed a bulky uterus with an endometrial thickness of 21 mm (Figure 1).

**Figure 2:**
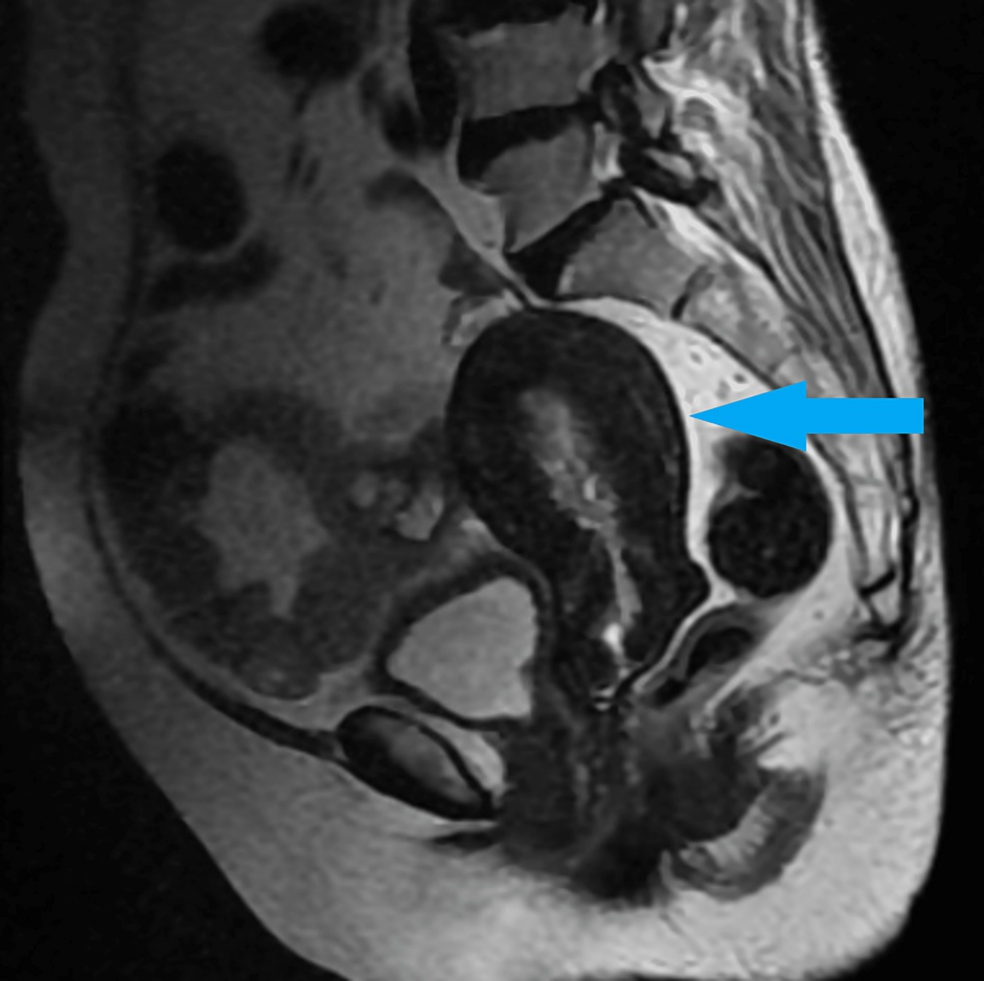
Magnetic resonance image showing a sagittal section of uterus (blue arrow)

**Figure 3:**
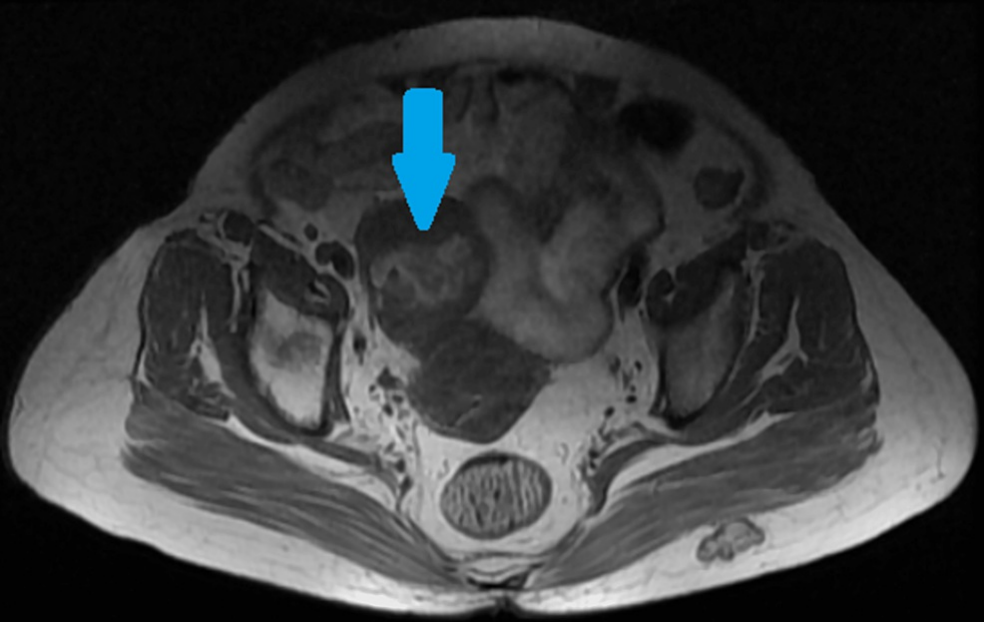
Magnetic resonance image showing an axial cut of the uterus with neoplastic ovarian mass (blue arrow)

Dilatation and curettage were performed in view of postmenopausal bleeding and thickened endometrium, which revealed endometrial type I cancer. Magnetic resonance imaging was carried out in view of co-existing endometrial cancer and an adnexal mass, and the results showed an endometrial mass measuring 21 x 14 mm in size. No evidence of involvement of the outer myometrium. A solid mass in the right ovary of size 58 x 61 mm with a hemorrhagic area within it was found (Figures 2, 3). Both sides of the pelvis have small, 6-7 mm lymph nodes that have not been much enhanced. No omental deposits were visible.

**Figure 4:**
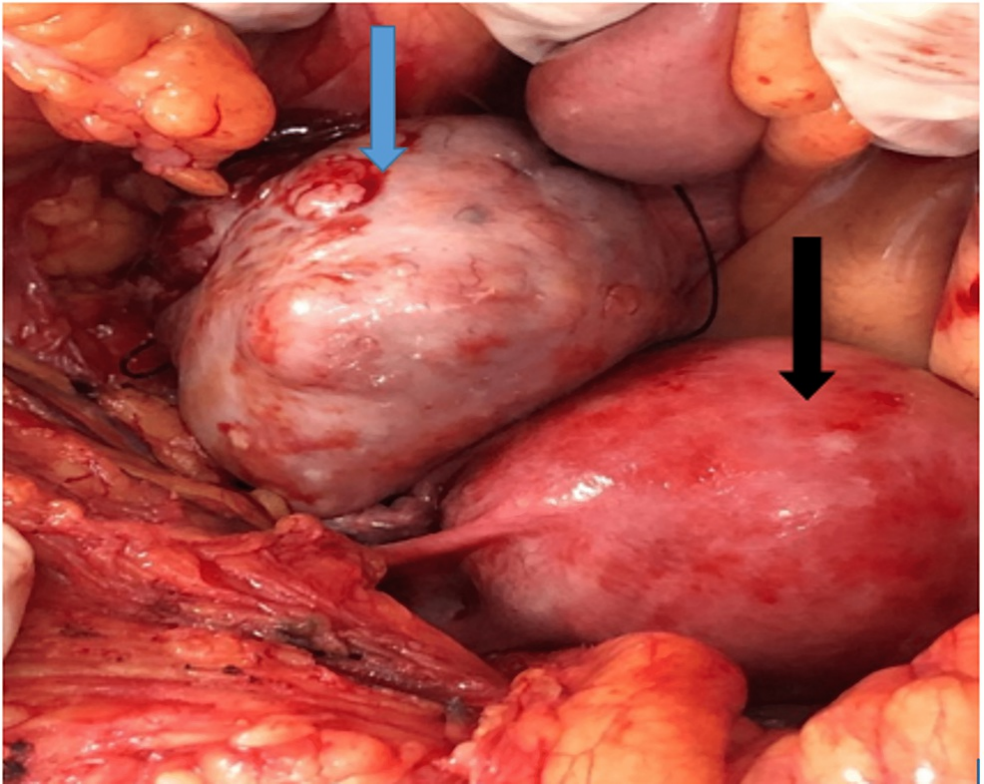
Right enlarged cystic ovary (blue arrow) with uterus (black arrow)

**Figure 5:**
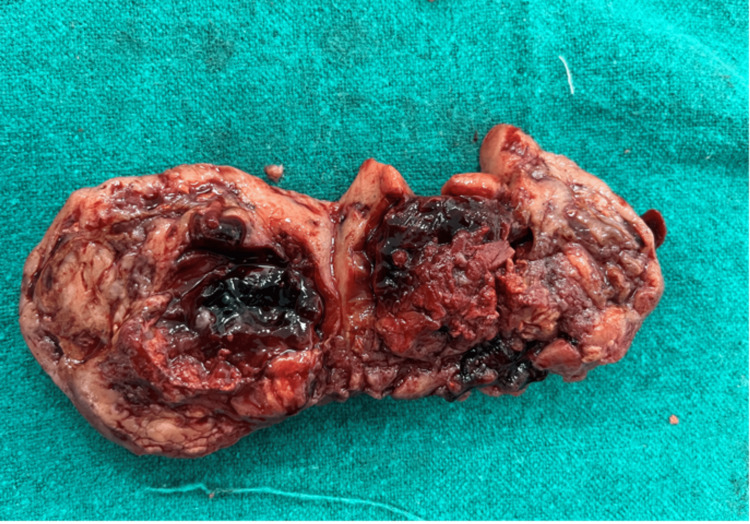
Cut section of the right ovary revealing cystic and hemorrhagic areas

**Figure 6:**
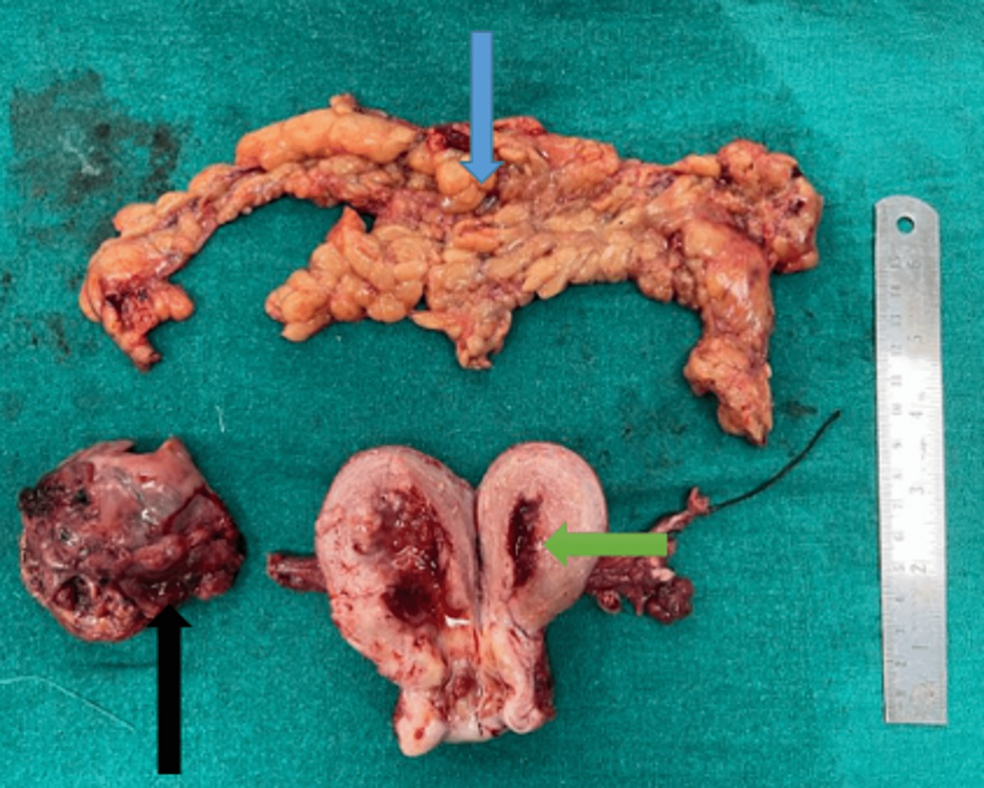
Cut section of uterus (green arrow) with right ovary (black arrow) with omentum (blue arrow)

The decision to perform a staging laparotomy was made based on the preliminary diagnosis of concurrent ovarian and endometrial cancer. The right ovary, which was 7 x 6 x 5 cm and had solid components with uneven margins and surfaces, as well as the uterus, were discovered during the patient's staging laparotomy (Figures 4, 5). There were no ascites, and the left adnexa was healthy. Cytology of peritoneal wash was done, which came to be normal. Upon examination, the omentum, pancreas, liver, and biliary tract were free of lesions. A modified radical hysterectomy (type B) was performed, along with bilateral pelvic lymph node dissections, low para-aortic lymphadenectomy, and supra-colic omentectomy (Figure 6). The specimens were then sent for histopathological analysis. She was discharged on the 12th post-operative day after suture removal and had a satisfactory postoperative recovery.

**Figure 7:**
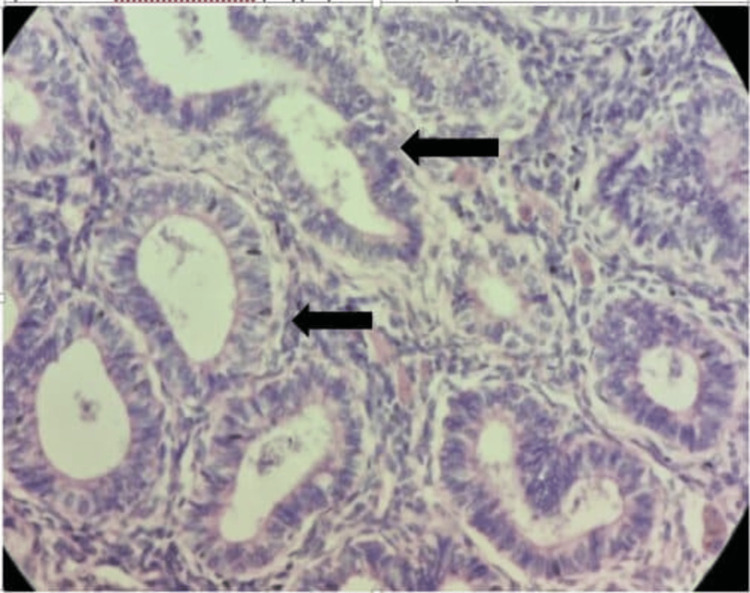
Histopathological cut section of endometroid carcinoma (Hematoxyline and Eosin stained, 40X), black arrows shows cells which are typically tall columnar with mild to moderate nuclear atypia

On histopathological examination, endometrium shows endometrial carcinoma (type I), myometrial invasion is less than 50%, and shows severe atypical endometrial hyperplasia with cystic hyperplasia without lymph vascular permeation. Lower uterine segment, cervix, left adnexa, bilateral pelvic lymph nodes, peritoneal biopsies, and omentum were free of tumours. It was graded stage IA based on the International Federation of Gynecology and Obstetrics (FIGO) classification (Figure 7).

**Figure 8:**
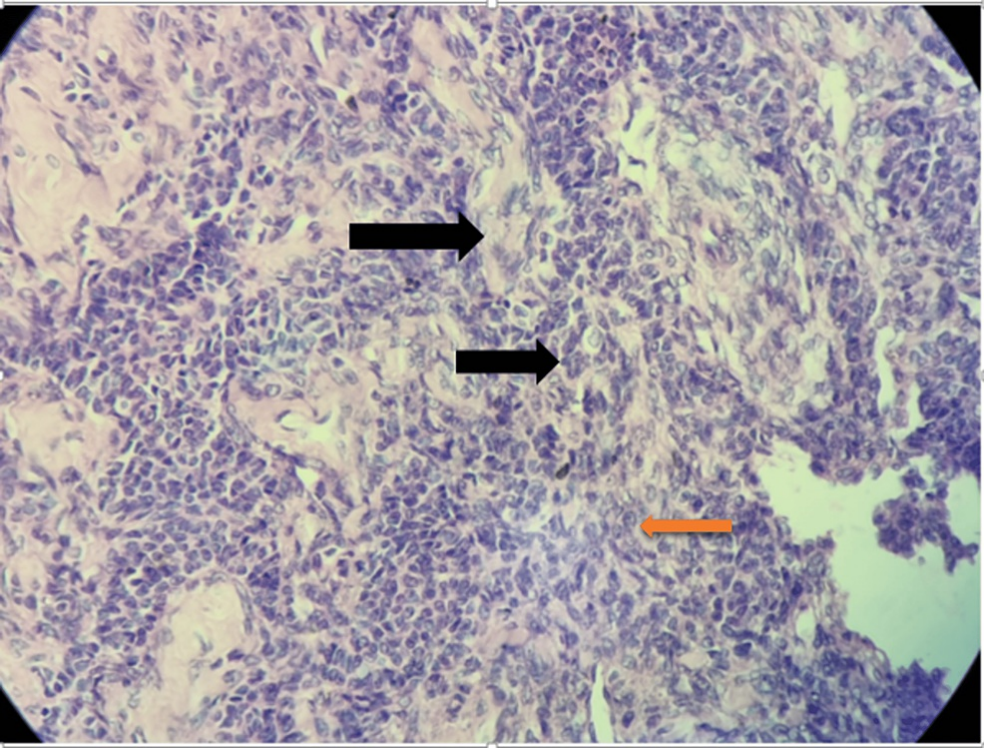
Histopathological cut section of granulosa cell tumor of ovary (Hematoxyline and Eosin stained, 40X), black arrows shows call exner body and orange arrow shows nuclear groove

The right ovary, measuring 7 x 6 x 5 cm, and the cut section showed cystic and hemorrhagic areas suggestive of adult granulosa cell tumor (Figure 8). The granulosa cells have tiny, whitish, round, or oval appearances. Additional histologic analysis showed fibroblasts, stromal, thecal, and coffee bean groove nuclei. Call exner bodies, which are collections of fluid and debris, are another important aspect. Immunohistochemistry for vimentin, inhibin, calretinin, and CD56 was positive.

The final diagnosis was stage 1A endometrial type I adenocarcinoma with adult granulosa cell tumor. Therefore, neither chemotherapy nor adjuvant radiotherapy were suggested. The patient no longer had vaginal bleeding at the two-week follow-up appointment following surgery. Her abdominal wound had fully healed, and her vital signs were normal. She had no new concerns when she was last examined two months after the operation.

## Discussion

Thecoma and ovarian granulosa cell tumors are the two most prevalent ovarian tumors that secrete estrogen; both are regarded as feminizing mesenchymomas of the ovary. Granulosa cell tumors make up 70% of all ovarian SCSTs. The adult form, which accounts for 95% of cases, and the juvenile type, which accounts for 5% of cases, are the two clinically and histologically distinct kinds. Related to this estrogen excess, coexisting pathology such as endometrial hyperplasia or adenocarcinoma has been found in 25 to 30 percent o patients with adult granulosa cell tumors [3]. The main changes in pre-pubertal patients are iso-sexual pseudo-precocity and the early development of secondary sexual features, whereas menometrorrhagia is the most prevalent symptom in adults and amenorrhea only occurs in rare instances. Ovarian SCSTs do occur more frequently than by chance in combination with a number of recognized genetic diseases. Ollier disease, which is characterized by numerous benign but unsightly cartilaginous neoplasms, and Peutz-Jeghers syndrome, which is characterized by intestinal hamartomatous polyps, are two associated illnesses. The adult tumor sub-type causes diffuse pelvic pain, breast tenderness, and vaginal hemorrhage most frequently in postmenopausal women [1-4].

**Figure 9:**
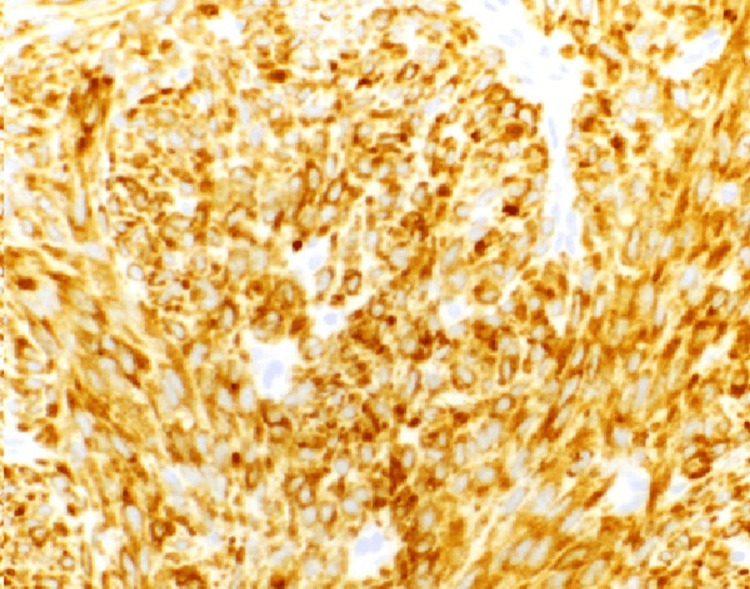
Inhibin A staining of granulosa cell tumor of ovary (inhibin A stain 40X)

**Figure 10:**
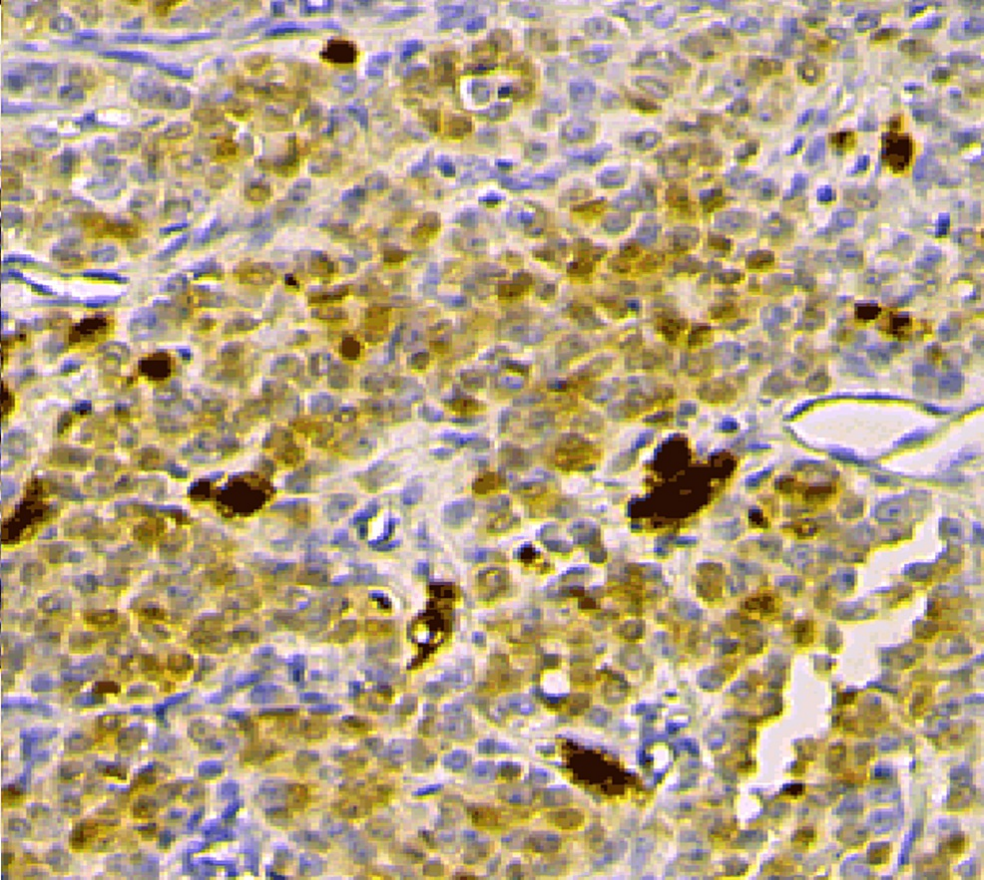
Calretinin staining of granulosa cell tumor of ovary (calretinin stain 40X)

Inhibin A (Figure 9) is useful as an immunomarker to distinguish sarcomatoid adult granulosa cell tumor from other spindle cell neoplasms because none of the other sarcomas tested expressed inhibin, except for one endometrial stromal sarcoma (ESS), which showed focal, weak cytoplasmic inhibin expression limited to sex cord-like areas. Calretinin appears to be less specific than inhibin because focal, strong calretinin immunoreactivity was identified in 11 leiomyosarcomas and one gastrointestinal stromal tumor (Figure 10). In addition, the case of endometrial stromal carcinoma with sex cord-like areas showed strong immunoreactivity for calretinin limited to the sex cord-like areas [6-8]. Only the area around granulosa cells is stained by reticulin.

Epithelial cell tumors, germ cell tumors, or sex cord-stromal cell tumors are three possible manifestations of ovarian cancer [1]. Germ-cell ovarian cancers are categorized as granulosa cell tumors. Both benign and malignant ovarian germ cell tumors are possible. Only 2% to 5% of ovarian malignancies are malignant ovarian germ cell tumors, which are extremely uncommon. However, throughout the second and third decades of life, they are the most prevalent type of ovarian cancer. According to the type of cell from which they are created, all germ cell cancers derive from the ovary's germ cells (the oocytes) [6].

Two categories have been identified for endometrial cancer. About 80% of cases fall into the first group (type 1), which is characterized by well-differentiated tumors that manifest as localized illness. The outcomes for these patients are frequently positive. Excessive estrogen exposure is linked to the development of type 1 endometrial cancer. Obesity, nulliparity with a history of infertility, late menopause, diabetes mellitus, unopposed estrogen therapy, tamoxifen therapy, and the use of successive oral contraceptive tablets are risk factors for type 1 endometrial cancer. Any of these sources of excess estrogen stimulates the endometrial lining continuously, which can cause endometrial hyperplasia and possibly endometrial cancer [1]. Endometrioid adenocarcinomas are composed of neoplastic glands resembling those of the normal endometrium. Cells are typically tall columnar with mild to moderate nuclear atypia. They form glands that are abnormally crowded or "back-to-back". Gland cribriforming, confluence, and villous structures are also common. It is these architectural forms, with the associated disappearance of intervening stroma, that distinguishes well-differentiated endometrioid adenocarcinoma from complex hyperplasia. In contrast, type II cancers are serous or clear cell histology, have no precursor lesion, and portend a more aggressive clinical course.

The grade of the tumor affects how an endometrioid carcinoma appears under the microscope. The tumor's architectural structure, its nuclear characteristics, or both are used to assign grades. The degree to which the tumor is made up of solid masses of cells as opposed to clearly identifiable glands determines the architectural grade. The variance in nuclear size and shape, the distribution of chromatin, and the size of the nucleoli all affect the nuclear grade. Grade 1 nuclei feature equally distributed chromatin, are oval, and are somewhat expanded. Grade 3 nuclei have strong eosinophilic nucleoli and are noticeably larger and pleomorphic, with irregular coarse chromatin [7].

Tumors should be evaluated using both architectural and nuclear criteria, according to the most recent revisions of the World Health Organization Histopathologic Classification of Uterine Carcinoma and the International Federation of Obstetricians and Gynecologists (FIGO) Staging System. When grade 3 nuclei, which are considered to be "notable" nuclear atypia, are present, the grade of tumors that are structurally grade 1 or 2 may be raised by one grade [7].

This was the case with our patient, who had a stage 1A International Federation of Gynecology and Obstetrics (FIGO) disease and a well-differentiated endometrioid adenocarcinoma. Since no adjuvant therapy was required, surgical management alone was suggested, and her follow-up examination six weeks following surgery was satisfactory.

## Conclusions

Endometrial carcinomas and adult granulosa cell tumors of the ovary seldom co-occur. Making this diagnosis requires a high degree of suspicion in addition to good imaging and histopathological examinations.
